# Plausibility Judgments of Atypical Symptoms Across Cultures: an Explorative Study Among Western and Non-Western Experts

**DOI:** 10.1007/s12207-017-9294-6

**Published:** 2017-07-28

**Authors:** Irena Boskovic, Douwe van der Heide, Lorraine Hope, Harald Merckelbach, Marko Jelicic

**Affiliations:** 1 Forensic Psychology Section, Maastricht University, P.O. Box 616, 6200 MD Maastricht, The Netherlands; 2 University of Portsmouth, Portsmouth, UK; 3 GGZ Centraal, Ermelo, The Netherlands

**Keywords:** Symptom validity assessment, Atypical symptoms, Cross-cultural research, Structured Inventory of Malingered Symptomatology

## Abstract

Symptom validity tests (SVTs) are predicated on the assumption that overendorsement of atypical symptoms flags symptom exaggeration (i.e., questionable symptom validity). However, few studies have explored how practitioners from different cultural backgrounds evaluate such symptoms. We asked professionals working in Western (*n* = 56) and non-Western countries (*n* = 37) to rate the plausibility of uncommon symptoms taken from the Structured Inventory of Malingered Symptomatology (SIMS), dissociative symptoms from the Dissociative Experience Scale (DES-T), and standard symptoms (e.g., anxiety, depression) from the Brief Symptom Inventory-18 (BSI-18). Western and non-Western experts gave similar plausibility ratings to atypical, dissociative, and standard symptoms: both groups judged BSI-18 symptoms as significantly more plausible than either dissociative or atypical symptoms, while the latter two categories did not differ. Our results suggest that the strategy to detect symptom exaggeration by exploring overendorsement of atypical items might work in a non-western context as well.

When a person is presenting with atypical mental problems (e.g., “my headaches are so severe that my feet hurt”), this may raise the suspicion of malingering. Malingering is defined as the intentional production of false or grossly exaggerated symptoms, motivated by external incentives. Such incentives may involve financial rewards, compensation, or reduced legal responsibility (American Psychiatric Association, 2000). However, what is considered to be an atypical symptom may depend on the cultural background of patients and evaluators (e.g., Weiss & Rosenfeld, 2012). Thus, cultural backgrounds may affect how patients express psychological or medical complaints and how healthcare professionals evaluate the plausibility of these complaints (e.g., Thakker & Ward, 1998; Kleinman & Cohen, 1997; Hausotter & Schouler-Ocak, 2007). Surveys suggest that professionals from different countries only moderately agree in their evaluation of mental disorders (Giosan, Glovsky, & Haslam, 2001) and neuropsychological symptoms (e.g., mild head injury; Ferrari, Constantoyannis, & Papadakis, 2001). Exaggerated symptoms might be more acceptable or even expected in one culture, but possibly an instant red flag for malingering in others (Charles, Gafni, Whelan, & O’Brien, 2006). Furthermore, the language in which a medical or psychological examination is conducted may affect the response style of patients (Harzing, 2006; Johnson, Kulesa, Cho, & Shavitt, 2005), leading to possibly inaccurate conclusions about significantly different prevalence levels of exaggerated symptomatology across countries (Nijdam-Jones & Rosenfeld, 2017). However, there are only a few cross-cultural studies on symptom validity assessment (e.g., Merten & Rogers, 2017), and even less research has focused on practitioners’ judgments of atypical symptoms across cultures.

Symptom validity tests (SVTs) aim to detect an exaggerated response style in patients (e.g., Larrabee, 2012). Many SVTs are predicated on the assumption that endorsing a relatively high number of atypical symptoms is indicative of symptom exaggeration. One widely used instrument (e.g., Martin, Schroeder, & Odland, 2015) is the Structured Inventory of Malingered Symptomatology (SIMS; Widows & Smith, 2005), which lists 75 bizarre, uncommon, atypical, and rare symptoms such as “There is a constant ringing in my ear” and “The voices that I hear, have never stopped since they began”. Endorsing more than 16 of these atypical symptoms indicates a heightened probability of exaggerated symptom presentation (Merckelbach & Smith, 2003). Although the internal consistency of the SIMS is satisfactory, its test-retest stability is sufficient, and its ability to discriminate between symptom exaggeration and honest responding is fairly effective (with sensitivities varying between 0.75 and 100%; van Impelen, Merckelbach, Jelicic, & Merten, 2014), some authors have expressed concerns about using SVTs such as the SIMS in patients or defendants with a non-Western background (Merten & Rogers, 2017; Nijdam-Jones & Rosenfeld, 2017). Specifically, Merten and Rogers (2017) note that the detection of exaggerated symptoms in minority groups might be complicated by culturally distinct illness expression and clinicians’ stereotypes about malingering in migrant workers.

Whether bizarre or atypical symptoms of the SIMS are also bizarre and unlikely in a non-Western context is an empirical question. Some scholars have speculated that Eastern cultures focus more on the somatic manifestations of psychiatric conditions such as post traumatic stress disorder (PTSD), while in Western countries patients emphasize the psychological impairments that accompany this condition (Kleinman & Cohen, 1997; see also Dückers, Alisic, & Brewin, 2016; Terheggen, Stroebe, & Kleber, 2001). This would suggest that different thresholds across cultures might apply in detecting atypical symptomatology. On the other hand, Van der Heide and Merckelbach (2016) compared SVT outcomes of several groups of asylum seekers who stayed in a psychiatric facility. Their study involved the following groups: (1) asylum seekers who had incentives to exaggerate their mental problems; (2) asylum seekers who did not have such incentives; (3) asylum seekers with a poor proficiency in the language of the host country (Dutch), and (4) asylum seekers with a good proficiency in Dutch. The authors compared these groups with regard to their endorsement of atypical symptoms taken from the SIMS. Atypical symptom endorsement occurred on a nontrivial scale and was related to incentives rather than language proficiency. In line with this, Nijdam-Jones and Rosenfeld (2017) concluded in their recent meta-analysis on cross-cultural feigning assessment involving 34 different tools that of the four psychiatric symptom validity measures (i.e., M-FAST, MENT, PAI, and SIMS), the SIMS had the highest overall classification accuracy, indicating the lowest level of variability across cultures and languages.

Unfortunately, no research has examined the cultural background of professionals who make decisions about the plausibility of various symptoms. With this in mind, we wanted to explore possible cultural variations in perception of atypical symptoms among professionals from Western and non-Western countries. Besides atypical symptoms, we also included common psychological problems such depression and anxiety. Furthermore, we included dissociative symptoms because they might overlap with atypical symptoms (Merckelbach et al., 2015). We anticipated that experts with a Western background would find the common psychological problems more plausible than atypical symptoms taken from the SIMS, with dissociative symptoms occupying an intermediate position. We had no a priori hypothesis about the symptom plausibility rank order of experts with a non-Western background.

## Method

### Sample

Our study included a convenience sample of 93 professionals from 22 countries. The average working experience of the professionals was 9.55 (SD = 8.43) years, 11 years (SD = 9.45) for Western and 7 years (SD = 5.50) for non-Western professionals (*t* (91) = 1.91, *p* < .05). The majority of them (72%) were working in the field of clinical psychology and psychotherapy, while 23% had medicine as their work setting. Western and non-Western professionals were mostly working in a clinical (43 and 32.5%, respectively), forensic (35.7 and 13.5%, respectively), or therapy (14.3 and 43.2%; respectively) setting. Groups did only differ with regard to the latter setting; Mann-Whitney *U* test = 774.00, *z* = −2.16, and *p* = .03.

Following Huntington (1993),^1^ we assigned professionals from North America, Western Europe, Australia, and New Zealand to the Western group. Professionals from East and South Europe, Asia, and Africa formed the non-Western group. In total, the Western group consisted of 56 professionals (60%), while the non-Western group consisted of 37 professionals (40%) (see Table 1).

**Table 1:** Frequencies of professionals from Western and non-Western countries

Country	Western	Non-Western
Australia	2	0
Bosnia and Herzegovina	0	1
Canada	4	0
China	0	3
Croatia	2	0
Germany	5	0
Greece	0	4
Indonesia	0	1
Ireland	1	0
Italy	7	0
Japan	0	1
Lebanon	0	2
Lithuania	6	0
Malaysia	0	2
Netherlands	8	0
New Zealand	1	0
Rwanda	0	1
Serbia	0	19
South Africa	0	2
United Kingdom	8	0
USA	12	0
Vietnam	0	1
Total	56	37

### Measures

We included the 37 items from the short form of the *Structured Inventory of Malingered Symptomatology* (SIMS; Smith & Burger, 1997; Malcore, Schutte, Van Dyke, & Axelrod, 2015 2), 8 items from the *taxon subscale* of the Dissociative Experiences Scale (DES; Bernstein & Putnam, 1986; Waller, Putnam, & Carlson, 1996), and 18 items from the *Brief Symptom Inventory-18* (BSI-18; Derogatis, 2001)*.* The SIMS items allude to psychological and neuropsychiatric symptoms that are, at least in a Western context, uncommon (Merckelbach & Smith, 2003). An illustrative item is: “Sometimes my muscles go limp for no apparent reason so that my arms and legs feel as though they weigh a ton”. The symptoms of the DES-taxon include the most pathological forms of dissociation and involve unusual phenomena such as “I have experienced being in a place and having no idea how I got there”. The items of the BSI-18 refer to the typical symptoms of depression and anxiety that are, at least in a Western context, relatively prevalent. Illustrative items are “Nervousness or shakiness inside “and “Difficulty in breathing”. All symptoms (37 + 8 + 18 = 63) were reformulated into statements of patients (e.g., “I have difficulty breathing”), mixed, and then presented to the professionals.

### Procedure

The study was conducted using Qualtrics. Participants were contacted via email, at medical and psychological conferences, or personally invited via email to join the study. Professionals first completed a set of demographic questions (e.g., work experience and field of work) and then asked to imagine a patient who is presenting with a specific symptom. The task of professionals was to grade each symptom on a 5-point plausibility scale (anchors: 1 = definitely authentic; 5 = definitely exaggerated).

After professionals had rated the 63 symptoms, they were asked questions about prevalence issues (“How often do you think patients exaggerate symptoms?”) using a 4-point scale (anchors: 1 = never; 2 = almost never, 3 = rarely, 4 = often), whether there are any clear signs for detection (anchors: “Yes” and “No”), and to provide a description of clues they considered to be important for the detection of exaggerated symptomatology.

#### Mean Plausibility Scores

We calculated the mean plausibility scores for SIMS, DES-taxon, and BSI-18 symptoms, separately (sum score/number of items). Thus, mean plausibility scores varied between 1 (definitely authentic) and 5 (definitely exaggerated). The data and the analysis can be found on Open Science Framework platform, following the link: https://osf.io/f8pqk/.

## Results

### Group Differences in Symptom Plausibility

We conducted a 2 (Western vs non-Western) × 3 (SIMS versus DES-T-versus BSI-18 items) analysis of variance (ANOVA). The main effect of cultural background was non-significant, *λ* = .98, *F* (3, 89) = 1.30, *p* = .28, which indicates that this factor did not affect how practitioners judged the plausibility of symptoms.^3^ Mean scores of Western and non-Western professionals for the three categories of symptoms are presented in Table 2. The table shows that the results were not significant with respect to any of the three measures used.

**Table 2:** Mean plausibility scores of Western and non-Western professionals

Measures	Groups	No.	*M* (SD)	*t* (91)	*p*
SIMS	Western	56	2.61 (.57)	1.85	.07
	Non-Western	37	2.34 (.84)		
	Total	93	2.50 (.70)		
DES-T	Western	56	2.54 (.71)	1.62	.10
	Non-Western	37	2.26 (.89)		
	Total	93	2.43 (.80)		
BSI-18	Western	56	1.80 (.84)	.52	.60
	Non-Western	37	1.73 (.71)		
	Total	93	1.77 (.64)		

*SIMS* Structured Inventory of Malingered Symptomatology (SIMS; Smith & Burger, 1997), *DES-T* taxon items of Dissociative Experiences Scale (DES; Bernstein & Putnam, 1986); *BSI-18* the Brief Symptom Inventory-18 (BSI-18; Derogatis, 2001)

### Rank Ordering Symptom Plausibility

We next ranked the average plausibility judgments of all symptoms for the Western and non-Western group, and calculated a correlation between groups’ rank orders. The Spearman rank correlation, *r*s = .91, *p* < .01, indicated high agreement between professionals’ judgment of items’ plausibility.

Using a series of *t* tests (with alpha values adjusted to .02), we explored whether professionals from Western and non-Western background differed in their plausibility judgments for individual symptoms. The groups evaluated three symptoms significantly different, all from the SIMS: Item 11 (“Recently I’ve noticed that my memory is getting so bad that there have been entire days that I cannot recall”), *t* (91) = 2.62, *p* = .01, *d* = .54; Item 12 (“At times I’ve been unable to remember the names or faces of close relatives so that they seem like complete strangers”), *t* (91) = 2.44, *p* = .017, *d* = .51, and item 19 (“Sometimes my muscles go limp for no apparent reason so that my arms and legs feel as though they weigh a ton.”), *t* (91) = 3.32, *p* < .01, *d* = .79. Western professionals evaluated these symptoms as less plausible (*M* = 3.17, SD = 1.17; *M* = 2.87, SD = 1.25; and *M* = 3.02, SD = 1.15, respectively) than the non-Western group (*M* = 2.47, *SD* = 1.40; *M* = 2.22, *SD* = 1.29; and *M* = 2.20, *SD* = 1.17, respectively). In Table 3, mean plausibility ratings and corresponding rank numbers can be found.

**Table 3:** Rank order of symptoms based on mean plausibility scores in the Western and non-Western group (from 1—highest plausibility to 63—lowest plausibility)

Items	Western rank	Western mean	Western SD	Non-Western mean	Non-Western SD	Non-Western rank
BSI-18—item 1	22	2.07	.91	1.92	.94	20
BSI-18—item 2	6	1.66	.64	1.48	.80	1
BSI-18—item 3	11	1.84	.76	1.75	1.06	13
BSI-18—item 4	18	1.98	.92	1.73	1.02	12
BSI-18—item 5	20	2.03	.97	1.95	1.02	23
BSI-18—item 6	15	1.89	.96	1.92	1.09	21
BSI-18—item 7	7	1.69	.91	1.75	1.06	14
BSI-18—item 8	13	1.86	.98	1.88	1.21	18
BSI-18—item 9	21	2.04	1.09	1.69	.92	10
BSI-18—item 10	4	1.64	.84	1.62	1.06	6
BSI-18—item 11	8	1.69	.76	1.56	.89	2
BSI-18—item 12	3	1.62	.75	1.61	1.01	3
BSI-18—item 13	2	1.59	.75	1.67	1.25	7
BSI-18—item 14	5	1.64	.77	1.61	.89	5
BSI-18—item 15	14	1.86	.86	1.84	.98	17
BSI-18—item 16	10	1.82	.92	1.67	1.18	9
BSI-18—item 17	16	1.91	1.05	1.92	1.09	22
BSI-18—item 18	1	1.58	.71	1.61	.82	4
DES-T—item 1	49	2.78	1.00	2.28	1.19	38
DES-T—item 2	48	2.75	1.10	2.40	1.27	46
DES-T—item 3	42	2.60	1.04	2.34	1.22	43
DES-T—item 4	45	2.62	1.16	2.43	1.25	48
DES-T—item 5	35	2.43	1.07	2.15	1.38	34
DES-T—item 6	32	2.41	1.02	2.15	1.11	33
DES-T—item 7	47	2.71	1.12	2.63	1.49	56
DES-T—item 8	19	2.00	1.04	1.70	1.15	11
SIMS—item 1	41	2.58	1.18	2.52	1.19	51
SIMS—item 2	12	1.85	.79	1.77	.93	16
SIMS—item 3	17	1.93	.97	2.06	1.13	27
SIMS—item 4	43	2.60	1.16	2.12	1.20	29
SIMS—item 5	37	2.47	1.15	2.05	1.38	26
SIMS—item 6	26	2.19	.90	2.14	1.10	30
SIMS—item 7	23	2.09	.94	1.89	1.12	19
SIMS—item 8	36	2.45	1.19	2.15	1.25	32
SIMS—item 9	9	1.78	.76	1.67	.97	8
SIMS—item 10	59	3.13	1.10	3.07	1.43	63
SIMS—item 11^a^	60	3.17	1.17	2.47	1.40	49
SIMS—item 12^a^	50	2.87	1.25	2.22	1.29	36
SIMS—item 13	25	2.18	.95	2.26	1.27	37
SIMS—item 14	28	2.30	.95	2.31	1.35	40
SIMS—item 15	57	3.09	1.19	2.94	1.24	62
SIMS—item 16	30	2.36	1.03	2.04	1.19	25
SIMS—item 17	31	2.37	1.13	2.07	1.49	28
SIMS—item 18	63	3.43	1.17	2.92	1.36	61
SIMS—item 19^a^	55	3.02	1.15	2.20	1.17	35
SIMS—item 20	33	2.41	1.09	2.14	1.15	31
SIMS—item 21	24	2.11	.94	1.96	1.18	24
SIMS—item 22	29	2.35	1.10	2.31	1.35	41
SIMS—item 23	53	2.98	1.26	2.89	1.24	60
SIMS—item 24	58	3.11	1.11	2.73	1.32	58
SIMS—item 25	39	2.55	1.11	2.37	1.39	44
SIMS—item 26	40	2.57	1.16	2.53	1.28	52
SIMS—item 27	54	2.98	1.18	2.39	1.29	45
SIMS—item 28	51	2.87	1.23	2.49	1.30	50
SIMS—item 29	61	3.18	1.14	2.67	1.37	57
SIMS—item 30	46	2.66	1.03	2.30	1.17	39
SIMS—item 31	27	2.23	1.09	1.76	1.19	15
SIMS—item 32	62	3.34	1.13	2.85	1.26	59
SIMS—item 33	44	2.61	1.09	2.54	1.30	53
SIMS—item 34	52	2.91	1.06	2.56	1.25	54
SIMS—item 35	38	2.48	1.04	2.42	1.21	47
SIMS—item 36	34	2.41	1.06	2.31	1.28	42
SIMS—item 37	56	3.02	1.21	2.61	1.33	55

*SIMS* Structured Inventory of Malingered Symptomatology (SIMS; Smith & Burger, 1997), *DES-T* taxon items of Dissociative Experiences Scale (DES; Bernstein & Putnam, 1986); *BSI-18* the Brief Symptom Inventory-18 (BSI-18; Derogatis, 2001)

^a^Adjusted alpha <.02

### Differences Between Symptom Categories

We compared the average plausibility judgments of SIMS, DES-T, and BSI-18 symptoms with each other, using paired *t* tests, in order to investigate whether practitioners would differentiate between atypical symptoms (SIMS), dissociative symptoms (DES-T), and common symptoms (BSI-18). SIMS (*M* = 2.50, *SD* = 0.70) and DES-T symptoms (*M* = 2.43, *SD* = 0.80) were evaluated as less plausible than BSI-18 symptoms (*M* = 1.77, *SD* = 0.64), *t* (91) = 11.63, *p* < .01 and *t* (91) = 8.85, *p* < .01. The difference in plausibility ratings for SIMS and DES-T symptoms was not significant: *t* (91) = 1.72, *p* = .09 (see Table 4).﻿

**Table 4:** Contrasts in plausibility judgment of SIMS, DES-T, and BSI items for full sample

Contrasts	Means (SD)	*t* (91)	*p*	Cohen’s *d*
SIMS–DES-T	2.50 (.70)	1.72	.09	0.16
	2.43 (.80)			
DES-T–BSI-18	2.43 (.80)	8.85	.001	.93
	1.77 (.64)			
BSI-18–SIMS	1.77 (.64)	11.63	.001	1.20
	2.50 (.70)			

*SIMS* Structured Inventory of Malingered Symptomatology (SIMS; Smith & Burger, 1997), *DES-T* taxon items of Dissociative Experiences Scale (DES; Bernstein & Putnam, 1986); *BSI-18* the Brief Symptom Inventory-18 (BSI-18; Derogatis, 2001)

### Prevalence and Clues of Symptom Exaggeration

The majority of professionals (64.5%) agreed that exaggeration is rare, and a quarter (24.8%) indicated that it occurs often, while other categories were less frequently chosen (Almost never: 7.5% and Never: 3.2%). Overall, prevalence estimates were related to plausibility ratings for SIMS (*r*s (93) = .30, *p* < .01), DES-T (*r*s (93) = .28, *p* < .01), and BSI-18 symptoms (*r*s (93) = .31, *p* < .01).

More than half of professionals believed that there are no clear signs of exaggerated symptomatology (57%), while 36.6% responded positive to this question, and the rest did not provide an answer (6.4%). Only 27% of the total sample, 37.5% of the Western group and 11% of the non-Western group, gave brief descriptions. In total, they generated 39 clues that were grouped into seven different categories: inconsistency within a report or an incongruence between reported symptoms and behavioral or anamnestic information (31%), over-reporting of implausible symptoms (18%), little or too specific (medical terminology) details of symptom reports (18%), presence of external benefits (13%), specific non-verbal clues (10%), individual factors such as educational background (7.5%), and presence of personality disorders (histrionic or antisocial) (2.5%).

## Discussion

Many SVTs are based on the rationale that overendorsement of atypical symptoms is reflective of symptom exaggeration. However, do atypical symptoms possess cross-cultural constancy? This question bears relevance to, for instance, the evaluation of asylum seekers with psychiatric problems, in which culturally shaped presentations of symptoms might be misjudged as feigning. This led some workers in the field to take a skeptical position as to the utility of SVTs across different cultural settings. For example, Merten and Rogers (2017; p. 106) wrote: “Assuming that any feigning measure is universally applicable across languages and diverse cultures is categorically unacceptable.” However, virtually no studies investigated whether professionals from various countries evaluate the plausibility of atypical symptoms in a similar way. With this in mind, we asked professionals working in Western and non-Western countries to judge a mix of symptoms that are—in a Western context—common or atypical.

Our findings can be summarized as follows: First, there were no significant overall differences between professionals from Western and non-Western countries in how they evaluated the plausibility of atypical symptoms (SIMS), dissociative experiences, (DES-T), and common mental problems such as depression (BSI-18). Both Western and non-Western professionals found BSI-18 symptoms more plausible than SIMS symptoms. This finding provides support for the review of Nijdam-Jones and Rosenfeld (2017) in which they concluded that the SIMS can be used to differentiate between exaggerating and non-exaggerating response styles in various language settings. Apparently, the atypical and bizarre nature of SIMS symptoms is constant across different cultural settings, which makes them useful for detecting an exaggerated symptom presentation.

Second, both groups regarded dissociative symptoms as less plausible than the common symptoms of the BSI-18. This might have to do with the fact that dissociative symptoms have a lower prevalence in the general population than symptoms such as depression and anxiety (Wittchen et al., 2011). However, professionals did not find dissociative symptoms more plausible than SIMS items. This observation is in line with recent research suggesting that in both healthy groups and clinical samples dissociative symptoms co-occur with symptom exaggeration (Merckelbach et al., 2015; Merckelbach, Boskovic, Pesy, Dalsklev, & Lynn, 2017). It might well be the case that individuals who engage in symptom exaggeration have a preference for dissociative symptoms because they regard these symptoms as indicating a profound impairment. For example, commenting on how malingering is portrayed in novels, Kuperman (2006; p. 70) concluded that “When madness is feigned, the eccentricity of simulation (…) sends a message to observers: ‘I’m not myself, so I’m not responsible’.” Thus, lay people may have the idea that dissociative symptoms (e.g., amnesia, depersonalization) compromise personal responsibility and in some settings (e.g., in court), this is precisely the impression that people would be motivated to convey.

Finally, the majority of professionals believed that exaggeration of symptoms occurs rarely. Inconsistencies between reported symptoms and behavioral or anamnestic information were seen as the most important clues for the detection of exaggeration. Both findings are in line with previous studies (Ruff, Klopfer, & Blank, 2016; Keesler, McClung, Meredith-Duliba, Williams, & Swirsky-Sacchetti, 2017).

A few limitations of the current study warrant comment. First, our study was based on a relatively small, convenience sample, symptoms were only provided in English, and we did not ask professionals to judge their English proficiency. Second, it might be the case that some of the professionals originally came from another country than the one they are currently working in. We did not obtained information as to their country of origin from all participants, but we assume that the cultural setting in which they presently work is more decisive for their evaluation of symptoms than the country in which they were born. Third, groups significantly differed in work experience and in the field of practice. However, even when we included work experience as a covariate, no differences were found. Fourth, and related to the previous point, the high agreement between Western and non-Western professionals in their plausibility ratings might reflect a common Western oriented training in psychology and/or medicine. Fifth and most importantly, our study focused on the plausibility of single symptoms, when in clinical practice, professionals will look at the combination of symptoms. Given these limitations, future studies may want to survey larger groups of Western and non-Western professionals in their own language, including those who had a non-Western training, and ask them to evaluate the plausibility of symptom combinations.

In sum, Western and non-Western professionals were found to show a high level of agreement in their evaluation of symptoms. Importantly, SIMS symptoms are seen by Western and non-Western professionals as bizarre, lending some credit to the use of the SIMS in non-Western groups (e.g., asylum seekers; Van der Heide & Merckelbach, 2016). Both groups of professionals also rated dissociative symptoms as less plausible than common BSI-18 symptoms. This might reflect a representative heuristic (Tversky & Kahneman, 1974), with BSI-18 symptoms being evaluated as more plausible than dissociative symptoms simply because the former are more prevalent than the latter. Alternatively, it might reflect the inherent problematic nature of dissociative symptoms due to the fact that malingerers have a preference for eccentric psychopathology among which dissociation (Merckelbach et al., 2017). Given this ambiguity, it might be wise to develop measures that tap into dissociative symptomatology, but that also include validity scales that correct for over-reporting.

Our results in no way imply that cultural differences in symptom presentation can be disregarded. It is important that clinicians inform themselves about such differences (see e.g., Young, 2014; Nijdam-Jones, Rivera, Rosenfeld, & Arango-Lasprilla, 2017; Weiss & Rosenfeld, 2012) and incorporate this knowledge in their diagnostic routines.

## Footnotes

1 Huntington proposed a differentiation between Western and non-Western civilizations primarily based on religion and economic and social development.

2 Following the appendix in the manuscript by Malcore et al. (2015), 38 items were excluded from the original SIMS version. This resulted in the final version of 37 items, which is not in accordance with authors’ claim that the final version includes 36 items. Our questions to the authors were left unanswered.

3 We also ran this ANOVA with working experience as a covariate, and none of the results reached significance: *λ* = .97, *F* (3.89) = .98, *p* = .40, SIMS *F* (1.90) = 2.71, *p* = .11, DES-T *F* (1.90) = 2.01, *p* = .16; BSI-18 *F* (1.90) = .30, *p* = .60.
